# Detection of right ventricular dysfunction by three – dimensional echocardiography and two - dimensional speckle tracking in breast cancer patients receiving anthracycline- based chemotherapy

**DOI:** 10.1186/s40959-023-00169-y

**Published:** 2023-04-06

**Authors:** Wafaa S. El-Sherbeny, Nesreen M. Sabry, Shaimaa B. El-Saied, Basma Elnagar

**Affiliations:** 1 Cardiovascular Medicine Department, Faculty of Medicine, Tanta University, Tanta, Egypt; 2 Clinical Oncology Department, Faculty of Medicine, Tanta University, Tanta, Egypt

**Keywords:** Three-dimensional echocardiography, Right ventricle, Cardiotoxicity, Longitudinal strain

## Abstract

**Background:**

Despite the cardiotoxic effect of anthracycline on the left ventricle (LV) was totally identified. The assessment of the anthracycline effect on the right ventricle(RV) by conventional echocardiography was a challenge due to its complex geometry. We aimed to evaluate the impact of anthracycline on the RV volume and function using 3 dimensional –echocardiography (3DE) and 2 dimensional -speckle tracking echocardiography (2D-STE) in patients with breast cancer.

**Methods:**

This prospective study was conducted on 66 female patients with breast cancer receiving anthracycline chemotherapy, in addition to full echocardiography, 2D-STE and 3DE evaluation of RV function and volume were done at baseline, after 4th cycle of chemotherapy, six and nine months after the end of chemotherapy.

**Results:**

Cardiotoxicity from anthracycline occurred in 18 patients whose LV ejection fraction became significantly reduced after 9 months of therapy according to that, the patients were divided into the non-cardiotoxic group (n:48) and the cardiotoxic group (n:18). At cardiotoxic group, 3D RV end-systolic volume, and 3D RV end-diastolic volume increased significantly at 6 months and continued till 9 months after the therapy end compared to baseline values (42.50 ± 5.98 vs. 50.44 ± 7.01, p = 0.005) and (86.78 ± 9.16 vs. 95.78 ± 9.23, p = 0.021).LV global longitudinal strain (GLS) showed a significant reduction early after 6 months of therapy, 2D GLS and free wall longitudinal strain (FWLS) of RV were significantly decreased at 6 months and continued till 9 months after therapy (-22.54 ± 0.79 vs. -19.53 ± 1.32, p = 0.001) and (-24.67 ± 1.27vs. -22.22 ± 1.41, p = 0.001) respectively. The variation of RV FWLS was a predictor of cardiotoxicity, the relative drop of RV FWLS > 19.3% had 83% sensitivity and 71% specificity, (AUC = 0.82) to identify patients who developed cardiotoxicity.

**Conclusion:**

3DE is a promising modality in recognizing the early changes in RV volumes and minute alteration in RV function and 2D-STE is a reliable predictor of RV systolic dysfunction which identify the subclinical affliction.

## Background

Breast cancer is shown to be the most common malignancy and plays a major role in death related to cancer worldwide amongst adult females in Global Cancer Statistics (GLOBOCA N) 2020 [1].

Anthracycline is considered the first-line drug according to the guidelines for the treatment of cancer breast and the category-recommended drug for neoadjuvant chemotherapy. It has a cardinal role in the postoperative adjuvant chemotherapy of breast cancer, with a powerful positive impact on breast cancer prognosis and survival. [2].

Though, anthracycline has toxic side effects such as myelosuppression and cardiotoxicity, especially cardiotoxicity with considerable clinical symptoms, this restricts its clinical use and influences the treatment course and the survival prognosis of patients with cancer [3]. The European Society of Medical Oncology [4] defined cardiotoxicity as the left ventricular ejection fraction (LVEF) dropping by ≥ 10% or to a value of ≤ 50%. Cardiotoxicity during anthracycline chemotherapy is dose-dependent; the higher cumulative dose is linked with a higher risk of cardiotoxicity [4].

While most studies have shown that early administration of angiotensin-converting enzyme inhibitors and beta blockers can result in LVEF regaining and a reduction in adverse cardiac events [5, 6], early detection of cardiotoxicity is required to prevent the progression to advanced heart failure (HF). Several studies have offered a wide range of echocardiography parameters to discover and predict anthracycline-induced left ventricle (LV) cardiotoxicity [7].

To the best of our knowledge, limited data are accessible regarding its effect on right ventricular (RV) function and regarding the parameters which could reflect subclinical RV dysfunction. Several studies addressed the importance of RV function as a strong predictor of prognosis in patients with LV systolic dysfunction [8, 9] and may predict subclinical LV dysfunction in animal models receiving anthracyclines [10].

The RV is characterized by its complex geometry and function; therefore, conventional two-dimensional echocardiography (2DE) has its limitations. Cardiac magnetic resonance (CMR) imaging is the gold standard in the evaluation of RV function and volumes; although it is costly, time-consuming, and inapplicable to every patient. [11, 12]

Three-dimensional echocardiography (3DE) is a new well-established modality that overcomes the 2D limitations and provides a precise assessment of RV volumes and functions in comparison to CMR. [13, 14]

Novel echocardiographic techniques, such as speckle tracking echocardiography (STE) for assessment of LV myocardial deformation, and global longitudinal strain (GLS) have been proven to identify subclinical LV cardiotoxicity [15]. Similar to the LV strain, the STE-derived RV longitudinal strain is feasible, reproducible, and prognostic in oncological patients [16, 17], it offers novel insights into RV function, some studies reported that RV strain deterioration preceded overt changes in its systolic function and the development of HF. [18, 19]

Our study was designed for the early detection of RV dysfunction using 3DE and myocardial longitudinal strain analysis in breast cancer patients receiving anthracycline chemotherapy.

## Methods

This prospective study was conducted on 73 female patients diagnosed with breast cancer who presented to the Clinical Oncology Department at Tanta University Hospital, from March 2020 to January 2022.

This study was conducted in the clinical oncology and cardiology departments at Tanta University Hospital. Privacy of all patients’ data was guaranteed, and every patient had a file with a private code number that included all investigations and kept the private data of every patient. Consent was obtained from all patients after a full clarification of the benefits and risks of treatment. Seven patients were lost during follow-up visits and hence excluded; 66 patients completed the whole study.

### Inclusion criteria

Female patients with breast cancer who had confirmed pathological biopsy, negative metastatic workup, Her2neu score negative, and adequate hematological, renal, and hepatic profiles.

### Exclusion criteria

Any patients with one of the following criteria were excluded from the study: non-sinus rhythm, history of ischemic heart disease or prior coronary intervention, any degree of LV systolic dysfunction at baseline study, significant valvular heart disease or prosthetic heart disease, poor echocardiographic window, chronic kidney disease, and diabetes mellitus. In addition, patients with any concomitant other chemotherapeutic agents, radiation therapy, or double malignancies, patients with severe malabsorption syndrome, and those with a performance status of more than 2 according to the WHO/ECOG (Eastern Cooperative Oncology Group) scores were all excluded from the study.

All patients were subjected to proper staging through a complete history, full clinical examination, and resting standard 12-lead surface electrocardiogram, in addition to a revision of all patients’ files. Surgical excision with axillary lymph node dissection. Biomarkers on tissue biopsy were done including the estrogen receptor (ER), the progesterone receptor (PR), proliferation marker Ki-67, and the human epidermal growth factor receptor-2 (HER2).

### Chemotherapy

All the patients received chemotherapy in the form of 4 cycles (anthracycline 60 mg/m2, IV day 1, Cyclofosamide 600 mg/m2 IV Day 1) repeated every 21 days. Followed by Paclitaxel 80 mg/m2 IV infusion weekly for 12 weeks.

### Imaging screening

Imaging screening was accomplished in all patients, including bilateral mamo-ultrasonography or bilateral breast ultrasonography (US), chest X-ray, computed tomography (CT) chest, pelvic abdominal US and/or triphasic CT abdomen and pelvis with contrast, and a bone scan (if indicated). If quarry metastatic lesions were suspected, 18 F-fluorodeoxyglucose positron emission myography would be performed.

### Echocardiographic data acquisition

The echocardiographic acquisition was performed using General Electric Vingmed Ultrasound Vivid E 9 system equipped with M3S transducer of 2–5 MHz for 2D and 4 V-D transducer of 1.5–4.0 MHz for 3D acquisition, and the images were sent to Echo Pack 2.02 for offline analysis. The echocardiographic examinations were done in the following 4 stages: before starting chemotherapy, after the 4th cycle of chemotherapy, and then at six and nine months after the end of chemotherapy.

Full 2D, M-mode, and Doppler echocardiographic examinations were done using the standard precordial views (apical 4-chambers, 2-chambers, 3- chambers views, long and short axis parasternal). Estimation of left ventricular volumes and function (EF%) by Simpson’s biplane method in 2D mode A 2D evaluation of RV function, including Tricuspid annular plane systolic excursion (TAPSE), was obtained via the M-mode method in the apical 4-chamber view and tissue Doppler parameters, especially peak tricuspid systolic velocity. FAC was calculated by tracing the RV endocardial contours at systole and diastole and applying the formula: 100 (RV end-diastolic area– . RV end-systolic area / RV end-diastolic area). Pulmonary artery systolic pressure (PSAP) was estimated using the continuous wave Doppler of the tricuspid regurgitation jet.

#### 2D LV global strain

The LV global strain (LVGLS) was assessed by GE AFI LV software from LV apical views (apical 3, apical 4, and apical 2, respectively) without foreshortening. At each view, two points were placed at the base and one at the apex. Then the software automatically tracked the LV contour. The measurements were approved after ensuring optimal tracking of the whole LV contour. LVGLS was automatically calculated as the averages of regional values from the 17 myocardial segments.

#### 2D RV strain

2D-STE - RV strain was measured offline using GE AFI RV software from RV-focused apical views, ensuring the capture of the whole RV without foreshortening using the RV automated function imaging software. After defining the region of interest (ROI) that involves the complete right ventricular myocardium and interventricular septum. Three points were placed at the basal RV free wall, the base of the septal wall, and the RV apex. Subsequently, the ROI automatically tracked the RV free wall and septal wall and divided them into (apical, mid, and basal segments). RV GLS was calculated from the average of all six segment values, and RV free wall longitudinal strain (RV FWLS) was obtained from the average of the three RV free wall segments.

#### 3D -echocardiography of RV

At the end of the 2D examination, RV 3D full-volume acquisition was attained from the RV-focused apical 4-chamber view using 4 or 6 consecutive beats to achieve adequate temporal resolution (26–40 volumes/s) during breath holding after adjusting gain, contrast, depth, and sector size, certifying complete RV visualization without stitching artifacts. The 4D Auto RVQ software was used for offline analyses. with the alignment of the RV vertical axis and its horizontal planes. Afterward, place six landmark points (two tricuspid annulus points and the RV apex point in the 4-chamber view, and the RV/LV posterior and anterior points plus the RV free wall point in the short axis mid view). Finally, the RV model was established with the possibility of editing if needed. 3D RV end-systolic volume (ESV), end-diastolic volume (EDV), stroke volume, and EF were calculated by the software.

### Statistical analysis

Data were analysed using Statistical Program for Social Science (SPSS) version 20.0 The mean standard deviation (SD) was used to express quantitative data.Qualitative data were expressed as frequency and percentage. The following tests were done:

- Independent-sample t-test of significance was used when comparing two means.
- A one-way analysis of variance (ANOVA) when comparing more than two means.
- Chi-square (X2) test of significance was used to compare proportions between two qualitative parameters.
- Pearson’s correlation coefficient (r) test was used for correlating data.
- ROC-curve: Receiver Operating Characteristic curve analysis.
- Regression analysis to influence one or more independent variables on a dependent variable.

## Results

This prospective study included 66 female patients diagnosed with cancer breast, who received their treatment in Clinical Oncology Department at Tanta University Hospital, and their cardiac assessment was done in the Cardiology department at Tanta University Hospital.

In our study, all the patients were her2neu - and received chemotherapy (4 cycles of anthracycline, followed by 12 weeks of paclitaxel).

The study was conducted on 66 patients, taking into consideration the cut–off value for cardiotoxicity definition (LVEF drop > 10% or to a value < 50%), and the patients were sub grouped into the cardiotoxic group (n = 18) and the non-cardiotoxic group (n = 48).The study was conducted on 66 patients, taking into consideration the cut–off value for cardiotoxicity definition (LVEF drop > 10% or to a value < 50%), and the patients were sub grouped into the cardiotoxic group (n = 18) and the non-cardiotoxic group (n = 48). As regard the patient characteristics in the Table (1) the mean age in the cardiotoxic group was (55.22 ± 5.76) while in the non-cardiotoxic group it was (44.13 ± 8.28) with a significant p-value.

**Table 1:** Demographic data of the studied groups

		Cardiotoxicity	Non-cardiotoxicity	Test	p. value
Stage	II (%)	8 (44.4%)	20 (41.7%)	X2: 0.041	0.839
	III (%)	10 (55.6%)	28 (58.3%)		
ER (%)		17 (94.4%)	45 (93.8%)	X2: 0.011	0.916
PR (%)		11 (61.1%)	34 (70.8%)	X2: 0.570	0.450
RTH (%)		16 (88.9%)	41 (85.4%)	X2: 0.134	0.714
HTN (%)		4 (22.2%)	17 (35.4%)	X2: 1.051	0.305
DM (%)		8 (44.4%)	13 (27.1%)	X2:1.819	0.177
Dyslipidemia (%)		7 (38.9%)	16 (33.3%)	X2:0.178	0.673
Age	Mean ± S. D	55.22 ± 5.76	44.13 ± 8.28	T: 5.223	0.001*
KI 67	Mean ± S. D	17.06 ± 7.02	17.31 ± 8.86	T: 0.111	0.912
Dose	Mean ± S. D	375.56 ± 36.66	363.33 ± 36.75	T: 1.204	0.233

ER: Estrogen receptor, PR: Progesterone receptor, RTH: Radiotherapy, and HTN: Hypertension, DM:diabetes mellitus

As regards the tumor stage, ER, PR, and the number of patients who received radiotherapy or hormonal therapy, there was no significant difference between both groups as in Table (1). The mean cumulative dose of anthracycline in the cardiotoxic group was (374.56 + 36.66), and in the non-cardiotoxic group (363.33 ± 36.75) with a non-significant Table (1). The cardiovascular risk factors, such as hypertension, diabetes, and dyslipidemia, showed no significant difference between the two groups.

### Standard echo parameter

All patients had normal LVEF at baseline, and at the end of chemotherapy, cardiotoxicity occurred in 18 patients (27.2%) during follow-up, 6 patients were diagnosed with cardiac dysfunction at 6 months after the end of therapy, and 12 patients developed cardiotoxicity after 9 months, LVEF was significantly reduced in the group after 9 months compared to baseline (62.44 ± 4.50 vs. 47.89 ± 3.88, p = 0.001).

### Right ventricle

The changes in the standard echocardiographic parameters at the beginning of chemotherapy and subsequent follow-up were demonstrated in Tables (2,3). Among the patients in the non-cardiotoxic group, there was no significant difference between the baseline and the follow-up parameters, Table (3). On the other hand, TAPSE and FAC started to decrease at 6 months and continued after 9 months from the end of chemotherapy in the cardiotoxic group.

**Table 2:** Echocardiographic parameters in the cardiotoxic group

Variables	Baseline			End			6 m.			9 m.			P1	P2
LVEF	62.44 ± 4.50			60.78 ± 4.51			58.89 ± 4.76			47.89 ± 3.88			0.085	0.001*
LVGLS	-21.21 ± 1.59			-20.37 ± 1.53			-17.49 ± 1.07			-15.22 ± 1.18			0.001*	0.001*
SPAP	14.11 ± 3.82			14.28 ± 3.37			16.44 ± 4.46			17.56 ± 5.36			0.374	0.088
RV-S	14.56 ± 1.79			13.78 ± 1.59			13.24 ± 1.73			13.04 ± 2.36			0.170	0.081
TAPSE	1.95 ± 0.19			1.94 ± 0.18			1.90 ± 0.11			1.82 ± 0.13			0.776	0.069
FAC	44.83 ± 4.44			44.50 ± 3.94			41.44 ± 4.23			38.00 ± 3.76			0.072	0.001*
RV EDV	86.78 ± 9.16			87.50 ± 8.97			95.78 ± 9.23			105.00 ± 8.98			0.021*	0.001*
RV ESV	42.50 ± 5.98			42.89 ± 5.92			50.44 ± 7.01			61.28 ± 8.45			0.005*	0.001*
RV EF	51.13 ± 3.09			51.12 ± 2.83			48.47 ± 3.59			43.82 ± 4.68			0.130	0.001*
RV FWLS	-24.67 ± 1.27			-24.62 ± 1.26			-22.22 ± 1.41			-19.23 ± 1.54			0.001*	0.001*
RV GLS	-22.54 ± 0.79			-22.44 ± 0.76			-19.53 ± 1.32			-17.86 ± 1.33			0.001*	0.001*

P1: Baseline values compared with 6 months

P2: Baseline values compared with 9 months

*: means significant

LVEF: left ventricle ejection fraction, LVGLS: left ventricle global longitudinal strain, SPAP: systolic pulmonary artery pressure, RV-S: right ventricle systolic wave, TAPSE: Tricuspid annular plane systolic excursion, FAC: fractional area change, RV EDV: right ventricle end-diastolic volume, RV ESV: right ventricle end-systolic volume, RV EF: right ventricle ejection fraction, RVFWLS: right ventricular free wall longitudinal strain, and RVGLS: right ventricular global longitudinal strain

**Table 3:** Echocardiographic parameters in the non-cardiotoxic group

Variables	Baseline			End			6 m.			9 m.			P1	P2
LVEF	62.46 ± 4.87			62.19 ± 4.85			61.60 ± 4.81			60.40 ± 5.10			0.829	0.171
LVGLS	-21.94 ± 1.25			-21.76 ± 1.26			-21.64 ± 1.22			-21.30 ± 1.33			0.657	0.068
SPAP	13.50 ± 3.59			14.77 ± 3.59			14.97 ± 4.06			15.46 ± 3.86			0.225	0.057
RV-S	13.96 ± 1.44			13.42 ± 1.50			13.25 ± 1.47			13.21 ± 1.72			0.108	0.084
TAPSE	1.87 ± 0.18			1.90 ± 0.16			1.87 ± 0.15			1.81 ± 0.11			0.990	0.230
FAC	45.25 ± 5.68			44.83 ± 5.38			44.10 ± 5.42			44.19 ± 5.72			0.743	0.785
RV EDV	89.02 ± 9.02			89.79 ± 8.77			92.79 ± 8.94			93.21 ± 9.38			0.175	0.108
RV ESV	42.67 ± 6.55			42.88 ± 6.65			44.85 ± 6.69			45.48 ± 7.27			0.394	0.181
RV EF	52.17 ± 4.68			52.37 ± 4.65			51.77 ± 4.42			51.87 ± 4.74			0.975	0.989
RV FWLS	-25.23 ± 1.63			-25.09 ± 1.62			-24.95 ± 1.66			-24.66 ± 1.81			0.842	0.356
RV GLS	-23.39 ± 0.85			-23.31 ± 0.85			-23.20 ± 0.87			-22.98 ± 1.03			0.753	0.123

P1: Baseline values compared with 6 months

P2: Baseline values compared with 9 months

LVEF: left ventricle ejection fraction, LVGLS: left ventricle global longitudinal strain, SPAP: systolic pulmonary artery pressure, RV-S: right ventricle systolic wave, TAPSE: Tricuspid annular plane systolic excursion, FAC: fractional area change, RV EDV: right ventricle end-diastolic volume, RV ESV: right ventricle end-systolic volume, RV EF: right ventricle ejection fraction, RVFWLS: right ventricular free wall longitudinal strain, and RVGLS: right ventricular global longitudinal strain

TAPSE wasn’t significantly decreased (p = 0.776 and 0.069 at 6 and 9ms); it only became slightly significant in 3 patients. FAC became significantly reduced at 9 months after the end of chemotherapy (p = 0.001). Tricuspid annular S` showed no significant differences during follow-up (p = 0.08), and no significant changes were observed in SPAP (p = 0.08), Table (2).

### 3DE and 2D-STE

In comparison with the baseline of the cardiotoxic group, there was a significant increase in both RVEDV and RVESV starting at 6 months (p = 0.021 and 0.005) and continuing after 9 months from the end of chemotherapy (both p = 0.001). However, the significant deterioration of RVEF was recognized at 9 months with a p-value of 0.001, mainly in 10 patients, Table (2), fig (a).

**Fig. 1:**
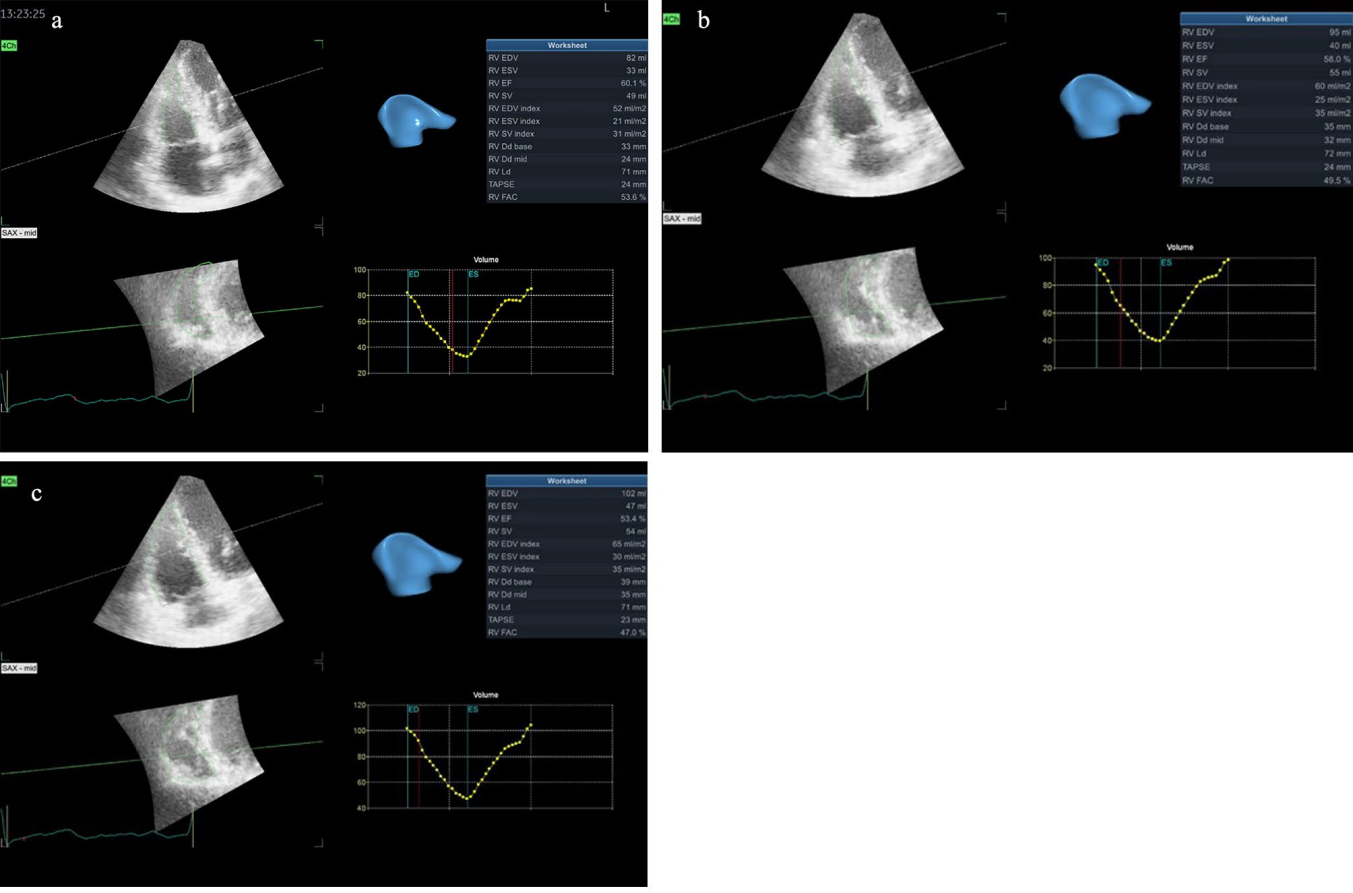
Three-dimensional echocardiography assessment of the right ventricle from RV-focused apical 4- chamber for a patient in the cardiotoxicity group using 4D Auto RVQ software delivering RV volumes and function after alignment of the RV axis and defining its landmarks at **(a)** baseline, **(b)** 6 months, and **(c)** 9 months after chemotherapy

LVGLS showed a significant reduction after 6 months of therapy and continued till 9 months in comparison with baseline values (-21.21 ± 1.5vs. -17.49 ± 1.07, p = 0.001 vs.-15.22 ± 1.18, p = 0.001), Table (2).

RV FWLS was reduced significantly after 6 months and continued to decrease after 9 months (-24.67 1.27 vs. -22.22 ± 1.41, p = 0.001 vs. 19.23 ± 1.54, p = 0.001) compared to baseline values. RV GLS showed a significant reduction at follow-up after 6 and 9 months compared to the baseline, Table (2), fig (b).

**Fig. 2:**
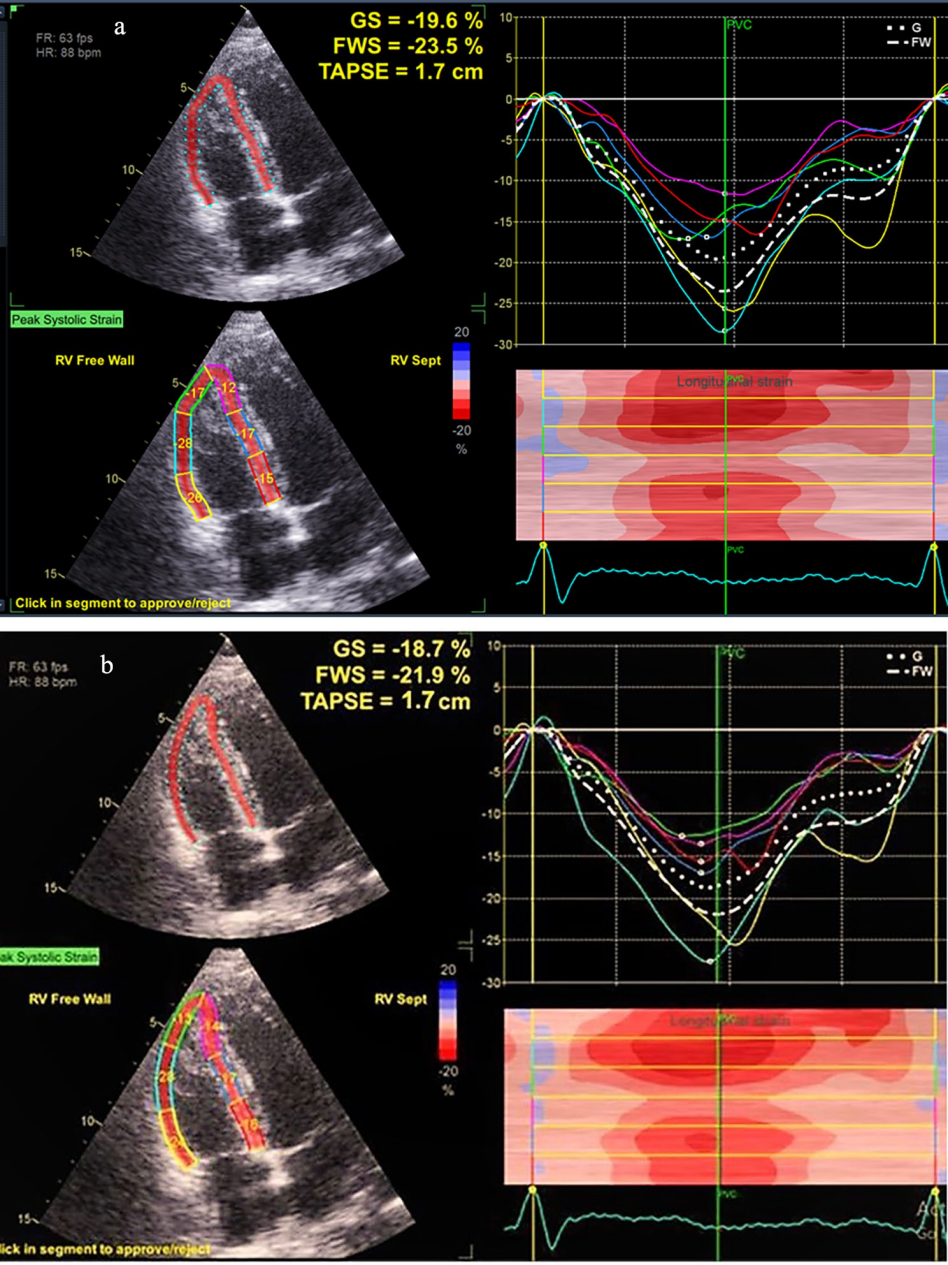
Tracking of the 6 – segment region of interest (ROI) in the endocardial border of the right ventricle (RV) from the apical four–chamber view was done. Then, RV free wall longitudinal strain(RVFWLS) was calculated by averaging peak systolic strain values of the three segments of the free wall, global longitudinal strain (GLS) was measured from the average values of peak systolic strain of RV free-wall and septal wall at **(a)** after 6 months and **(b)** after 9 months of chemotherapy

In our study, out of 18 patients who experienced cardiotoxicity, only 16 patients had an abnormal RV FWLS value after 6 months of chemotherapy.

The percentage variation between the baseline and follow-up values of the LVEF, LVGLS, RV FWLS, and RVGLS parameters was studied. Δ LVEF, Δ LVGLS, and Δ RV FWLS in proportions to the original value, a significant correlation was reported between the changes in LVGLS and RV FWLS at follow-up (r = 0.539, p = 0.018).

Δ LVGLS decreased by 25% to predict subclinical cardiotoxicity in our study, which was higher than the reported percentage (a reduction of 15% was enough to predict cardiotoxicity). The ROC curve analysis of the changes in RV FWLS established that a relative drop in RV FWLS > 19.3% after 6 months had an 83% sensitivity and a 71% specificity (AUC = 0.82) to identify patients who developed cardiotoxicity, fig (c).

**Fig. 3:**
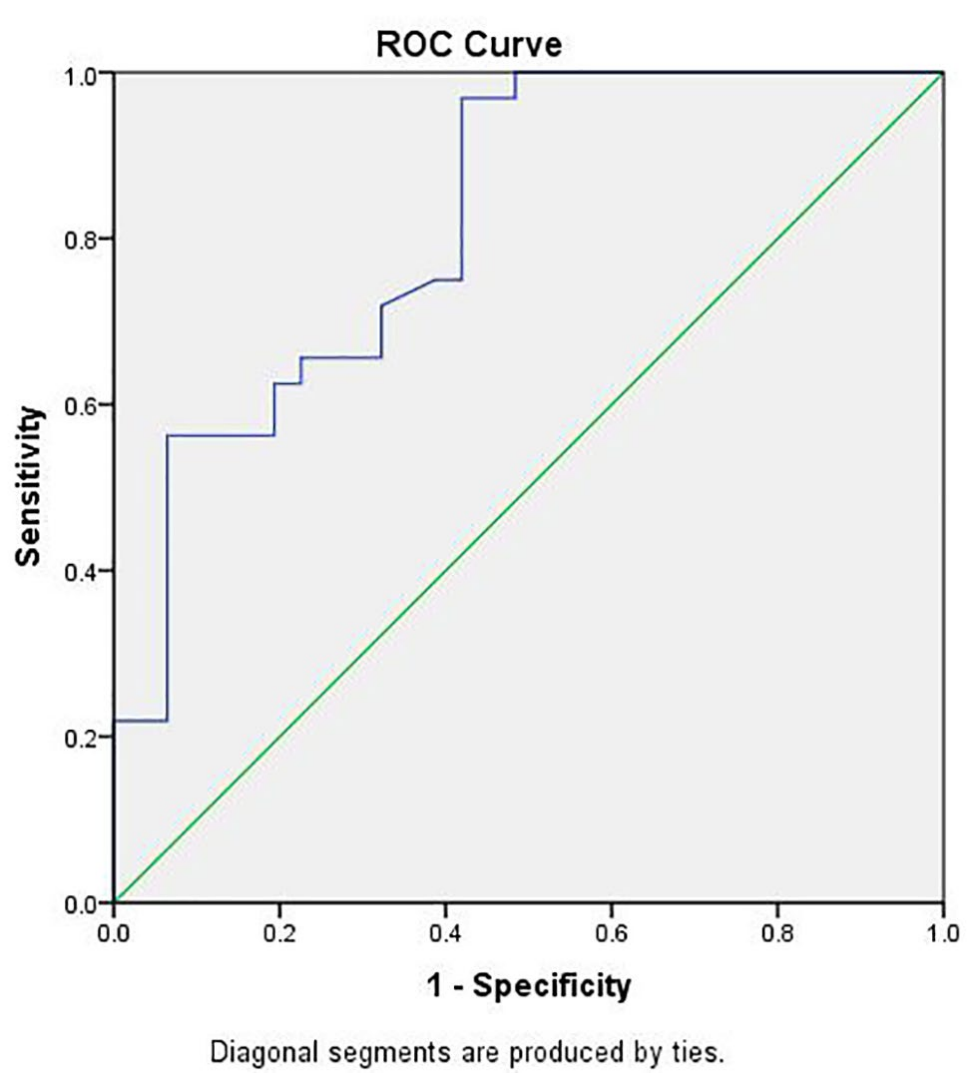
Receiver operating characteristic (ROC) curve analysis for percentage variations of right ventricular free wall longitudinal strain (RV FWLS) for prediction of cardiotoxicity, a relative drop of RV FWLS > 19.3% had a sensitivity of 83% and a specificity of 71% with areas under the curve (AUC) of 0.82

Furthermore, there was a significant association between the cumulative dose of anthracycline and the changes in RV FWLS (r = -0.806, p = 0.001).

Univariate regression analyses for the predictors of subclinical chemotherapy-induced cardiotoxicity (CTRCD) were done, showing the significance between the percentage variation for the following parameters ( Δ RV FWLS, Δ RV GLS, Δ LVGLS, Δ RVEDV, and ESV ) and the cumulative dose of anthracycline, Table (4). Also, a multivariate analysis of these variables showed that the RVFWLS and LVGLS were independent significant predictors of CTRCD on their own. Table (4).

**Table 4:** Univariate and multivariate analysis between the cumulative dose of anthracycline and the echocardiographic parameters in the cardiotoxic group

Variables	Univariate		Multivariate	
	OR (95% CI)	P value	OR (95% CI)	P value
∆ RVFWLS	0.484 (0.283–0.619)	0.001*	0.519 (0.319–0.759)	0.001*
∆ LVGLS	0.627 (0.218–0.894)	0.001*	0.725 (0.367–0.926)	0.009*
∆ RVEDV	1.512 (1.386–2.829)	0.036*	1.625 (0.237–1.805)	0.095
∆ RVESV	2.695 (1.365–4.841)	0.013*	1.828 (0.199–3.748)	0.081
∆ RVGLS	0.482 (0.296–0.748)	0.028*	0.734 (0.329–2.652)	0.102

∆ : changes in the measurements between baseline and 6 months after the end of therapy

LVGLS: left ventricle global longitudinal strain, RV EDV: right ventricle end-diastolic volume, RV ESV: right ventricle end-systolic volume, RVFWLS: right ventricular free wall longitudinal strain, and RVGLS: right ventricular global longitudinal strain

## Discussion

Anthracyclines are considered the most predominant cardiotoxic drug that generates reactive oxygen species and negatively affects cardiac tissues, leading to HF [20]. In fact, one meta-analysis showed that there was a higher risk of clinical and subclinical cardiotoxicity with anthracycline therapy than with regimens that didn’t have anthracyclines [21]. Despite the adverse negative consequences of anthracycline chemotherapy, it plays a key role in treating various types of cancers including 32% of breast cancer, 57–70% of geriatric lymphoma, and 50–60% of childhood cancer [22].

In this study, the effects of anthracyclines on LV function were assessed in 66 women with breast cancer.18 patients who developed cardiotoxicity had a significant deterioration in LVGLS early before the reduction in LVEF, Clinical, and subclinical anthracycline-related LV dysfunction had been well-researched in breast cancer patients. [23, 24]

However, RV dysfunction was not considered in the diagnosis of cardiotoxicity. The anthracycline-related toxic effects on the RV have become the focus of intense interest ,as the prognostic significance of RV dysfunction on the outcome of patients with HF has been established. [25, 26] The thinner structure of the RV with fewer myofibrils makes it susceptible to damage by chemotherapy, although the evaluation of the RV by conventional 2DE has a limited degree of accuracy. The CMR is the gold standard technique for the assessment of RV volume and function. Recent studies have confirmed the accuracy of 3DE-determined RV volumes and function. STE-derived RV longitudinal strain has been shown to be possible, repeatable, and prognostic in oncological patients. [16, 17] Our study aimed to assess the changes in RV function and volumes after chemotherapy using 3DE and to detect subclinical RV dysfunction using 2D-STE.

In the current study, the conventional RV echocardiographic parameters showed a slight reduction in FAC measurement that appeared late during the follow-up by 6 months, and this reduction increased to become significant at the end of 9 months after the chemotherapy. On the other hand, TAPSE did not show a significant reduction at the end of chemotherapy or through follow-up period. Similar studies reported a significant reduction in RV FAC during the period of chemotherapy and follow-up, even though the reduction was within the normal range. [27, 28] Other studies reported a significant change in TAPSE at the end of chemotherapy or during follow-up. [29, 30] Rui et al. [31]and Cherate et al. [32] did not detect a change in FAC or TAPSE in associate with subclinical RV deterioration at the end of chemotherapy even though, Tanindi et al. [33] and Xu et al. [34] showed a significant change at the end and after 12 of months the chemotherapy. Therefore, the debate in the evaluation of RV function by traditional methods leads to diminishing of their role in early subclinical RV affection, due to RV’s complex geometry.

The RVESV and RVEDV in the cardiotoxic group in our study statistically increased after the end of chemotherapy by 6 months and continued by 9 months. However, the increase in ESV was more significant than in EDV at 6 months from the end of chemotherapy (P = 0.005 vs. P = 0.021 respectively). RVEF decreased alongside with LVEF, and they started during the follow-up and reduced significantly after 9 months, 10 patients (15.1% of total patients) had a significant RV dysfunction (RVEF < 45%). The reduction in RVEF wasn’t accompanied by an increase in PASP (afterload), and this could be supported by the direct toxic effect of anthracycline on RV as well as LV. Similarly, Rui et al. [31] studied the effect of anthracycline on 74 patients and found that RVESV and RVEDV went up significantly during chemotherapy, followed by a reduction in RVEF at the end of chemotherapy.

Moreover, Wang et al. [30] reported a significant increase in RV volumes accompanied by an earlier decrease in RVEF at the end of chemotherapy and subsequently the reduction in LVEF later during the follow-up in 61 patients with B-cell lymphoma .In agreement with our results, Souza et al. [35] and Grover et al. [36] studied the anthracycline impact on RV using CMR in patients with breast cancer and demonstrated that the right ventricular remodelling induced by anthracycline in the form of myocardial oedema, a decrease in RV mass, and myocardial fibrosis with an increase in volumes, mainly ESV, followed by a decrease in RV function.

Our study reported in the cardiotoxic group, RV dysfunction was detected in 10 patients (55%) by RVEF, concomitantly with the reduction in LVEF, and detected in 16 patients (88%) by RV FWLS early after 6 months of therapy. Both RV FWLS and RVGLS significantly decreased after 6 months and continued to decrease after 9 months (p = 0.001) compared to baseline values. RV dysfunction evaluated by conventional parameters TAPSE and S` showed no significant changes.

In the same line with Vahabi et al. [37] who studied 62 patients to assess the decline in RV GLS, FAC as well as RV FWLS following anthracyclines treatment. The study showed that the RV FWLS and GLS were significantly lower in patients with cardiotoxicity than in patients with preserved LVEF. However, there was no statistically significant change in the 2D measures of RV function. Similarly, Planek et al. investigated the cardiotoxicity of doxorubicin in 35 lymphoma patients and demonstrated a significant deterioration in RV FAC, RV FWLS, as well as RV GLS after a 6-month follow-up. In contrast with our results, their study did not detect any significant reduction in LVEF after six months of follow-up and concluded that doxorubicin therapy is associated with subclinical RV dysfunction [28].

The study of Xu et al. [34] who assessed RV function in 95 women with breast cancer receiving epirubicin therapy, found that 3D GLS of the LV and both RV GLS and FWLS were significantly depressed at 12 months as the early sign even without the occurrence of cardiotoxic affection of LVEF and RVEF, these variations of the 3DRV strain allow the identification of initial subclinical RV dysfunction when conventional parameters are unaffected, similarly with our results.

Initially, anthracycline-associated cardiotoxicity was linked to some factors, including high cumulative doses and patients with a history of cardiovascular diseases. It was revealed that a dose of > 400 mg/m2 was associated with the highest risk of cardiac injury, with an early incidence of HF of approximately 3%, 7% at a dose of 550 mg/m^2^, and 18% at 700 mg/m2. Consequently, it was suggested that the maximum lifetime cumulative dose of anthracycline should not exceed 550 mg/m^2^ [38]. Then, it was decided to limit the cumulative anthracycline dose to 400 to 450 mg/m^2^ [39]. Based on the results of our study, the patients received four cycles of anthracycline followed by 12 weeks of paclitaxel. The mean cumulative dose of anthracycline in the cardiotoxic group was (375.56 ± 36.66), while in the non-cardiotoxic group it was (363.33 ± 36.75), with a non-significant difference. Furthermore, there was a significant negative association between the cumulative dose of anthracycline and the declining percentage in RV FWLS (r= -0.806; p < 0.001). On the contrary, various studies have reported a cardiotoxic effect that is associated with lower anthracycline doses. In Cajella and colleagues’ study [17], the former LVEF was associated with doxorubicin’s mean dose of 231 ± 19 mg/m^2^. Similarly, Planek et al. [28] reported a cumulative doxorubicin dose of more than 200 mg /m^2^ and found a significant deterioration in RV FAC, RV FWLS, and RV GLS. In fact, one study has exhibited 1.6 folds of getting HF at a dose of 300 mg/m^2^ [40].

There is no consensus in the literature regarding the RV GLS value that can predict cardiotoxicity; an essential argument in RV strain analysis is whether GLS or FWLS should be assessed. The ROC curve analysis for the changes in RVFWLS proved that the relative drop of RV FWLS > 19.3% had a sensitivity of 83% and a specificity of 71% (AUC = 0.82) to identify patients who developed cardiotoxicity, and RV FWLS had a higher predictive value than GLS for the identification of cardiotoxicity. Keramida et al. [41] observed that the cut-off value of RV GLS percent change that identified cardiotoxicity was 14.8% with a sensitivity of 66.7%, a specificity of 70.8%, and an AUC of 0.68 in breast cancer patients receiving trastuzumab.

Cherata et al. [32] who assessed RV systolic function in 68 cancer patients receiving cardiotoxic agents demonstrated that a 17% reduction of RV FWLS had a sensitivity of 55% and a specificity of 70% with an AUC of 0.75 to identify patients with CTRCD.

Another study reported that the optimal cut-off value of 17.5% of 3D RV FWLS percent changes showed high prognostic accuracy for subclinical cardiac dysfunction, with an AUC of 0.74, a sensitivity of 80.5%, and a specificity of 65.8% [34].

Our study found that longitudinal strain analysis can find subclinical RV dysfunction early even when the standard 2D indices of RV function are not changed. FWLS is more accurate than RV GLS in the evaluation of RV systolic function since the intraventricular septum is a constituent part of the LV.

## Conclusions

Longitudinal strain analysis by 2D STE allows the recognition of subclinical RV dysfunction when conventional indices of RV function are unaffected. 3DE is a simple and low-cost technique that is not inferior to CMR for the assessment of RV volumes and function. RV FWLS could predict subsequent cardiotoxicity in breast cancer patients receiving anthracycline chemotherapy. These new echocardiographic parameters could add a further prognostic assessment for cancer patients, especially those who are at high risk of CTRCD.

### Study limitations

Small sample size, short follow-up period, and in our institute, there is no routine follow-up for patients by echocardiography after their therapy has been completed, therefore the recovery of LV dysfunction is difficult to be estimated, and that recovery would be influenced in patients with reduced RV FWLS, so further studies are needed.

## Data Availability

The datasets used and analysed during the current study are available from the corresponding author on reasonable request.

## Abbreviations

LV: Left ventricle
EF: Ejection fraction
HF: Heart failure
RV: Right ventricle
2DE: Two-Dimensional echocardiography
CMR: Cardiac magnetic resonance
3DE: Three -Dimensional echocardiography
2D STE: Two -Dimensional speckle tracking echocardiography
GLS: Global longitudinal strain
ECOG: Eastern Cooperative Oncology Group
ER: Estrogen receptor
PR: Progesterone receptor
HER2: Human epidermal growth factor receptor
US: Ultrasonography
CT: Computed tomography
TAPSE: Tricuspid annular plane systolic excursion
FAC: Fractional area change
PASP: Pulmonary artery systolic pressure
ROI: Region of interest
RV FWLS: Right ventricle free wall longitudinal strain
ESV: End systolic volume
EDV: End diastolic volume
CTRCD: chemotherapy-induced cardiotoxicity
